# Publisher Correction to *EJNMMI Radiopharmacy and Chemistry* Volume 1 (2016)

**DOI:** 10.1186/s41181-018-0049-9

**Published:** 2018-11-26

**Authors:** BioMed Central

**Affiliations:** https://www.biomedcentral.com/

The metadata in the HTML format of these original articles (Eppard et al., 2017; Decristoforo & Patt, 2017; Domnanich et al., 2017; Chan et al., 2017; Jovalekic et al., 2017; Zhang & Villalobos, 2017; Meckel et al., 2017; Li et al., 2017; Colin et al., 2017; Koziorowski et al., 2017; Ooms et al., 2017; Elsinga, 2017; Seemann et al., 2017; Müller et al., 2017; Luurtsema et al., 2017) were published with an incorrect cover date. The correct cover date is December 2016. This does not alter the text of the original articles.

The publisher apologises for this processing error.
